# A Scatter-Based Prototype Framework and Multi-Class Extension of Support Vector Machines

**DOI:** 10.1371/journal.pone.0042947

**Published:** 2012-10-30

**Authors:** Robert Jenssen, Marius Kloft, Alexander Zien, Sören Sonnenburg, Klaus-Robert Müller

**Affiliations:** 1 Department of Physics and Technology, University of Tromsø, Tromsø, Norway; 2 Machine Learning Laboratory, Berlin Institute of Technology, Berlin, Germany; 3 Molecular Health GmbH, Heidelberg, Germany; 4 TomTom Research, Berlin, Germany; 5 Machine Learning Laboratory, Berlin Institute of Technology, Berlin, Germany; 6 Department of Brain and Cognitive Engineering, Korea University, Seoul, Korea; Institution of Automation, CAS, China

## Abstract

We provide a novel interpretation of the dual of support vector machines (SVMs) in terms of scatter with respect to class prototypes and their mean. As a key contribution, we extend this framework to multiple classes, providing a new joint Scatter SVM algorithm, at the level of its binary counterpart in the number of optimization variables. This enables us to implement computationally efficient solvers based on sequential minimal and chunking optimization. As a further contribution, the primal problem formulation is developed in terms of regularized risk minimization and the hinge loss, revealing the score function to be used in the actual classification of test patterns. We investigate Scatter SVM properties related to generalization ability, computational efficiency, sparsity and sensitivity maps, and report promising results.

## Introduction

Dualization is a critical step in support vector machines (SVMs) [1] and other kernel-based learning algorithms [2], [3], since the actual optimization, or training, is carried out in the dual space. Despite this, the algorithms are most often formulated and interpreted solely in terms of the primal optimization problem, e.g. in the context of regularized risk minimization. A rare exception is provided by the convex hull view of the dual of binary SVMs [4], [5]. This alternative view yields additional insight about the algorithm and has also lead to algorithmic improvements [6], including online training [7]. An extension from the binary case to the multi-class case has furthermore been proposed in [8]. The dual view therefore in this case provides a richer theory by complementing the primal view.

In this paper, we contribute a new view of the dual of binary SVMs (a short version of this work appeared in [9]). We concentrate on the so-called 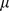-SVM [10], and interpret the dual in terms of *class prototypes*. Specifically, we cast the dual optimization problem as a minimization of *between-class scatter* with respect to the class prototypes and their arithmetic mean. This adds new intuition to the recent prototype framework of several binary kernel-based classifiers put forth in [11]. More importantly, we note that scatter is inherently a multi-class quantity. Our scatter-based view of the dual of SVMs therefore suggests a natural extension of the 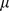-SVM to operate *jointly* on 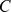 classes. Interestingly, this key contribution, which we fittingly refer to as Scatter SVM, does not introduce more variables to be optimized than the number 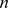 of training examples. In addition, the number of constraints are kept low due to the global reference point provided by the mean of the prototypes, at the order 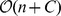 or 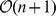 depending on whether or not a bias parameter is included in the problem formulation. This is a major computational saving compared to previous joint SVM approaches [12]–[15] which typically require optimizing 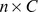 variables under a huge amount of constraints. Another prototype-based joint approach [16] also requires optimizing 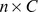 variables, although the number of constraints are much reduced compared to the aforementioned joint SVM methods. Non prototype-based approaches also exist, e.g. tree-based methods [17].

In fact, the number of optimization variables and constraints of Scatter SVM are at the same level as the binary counterpart. Binary SVMs are frequently used in practice, also to solve the multi-class prediction problem. Although this approach breaks a joint optimization problem into multiple independent binary problems [18], [19], it is often used since solvers based for example on sequential minimal optimization (SMO) provide a fast optimization for each one-vs.-rest or one-vs.-one problem. We develop an SMO-like dedicated and highly efficient optimization procedure for Scatter SVM, making our approach basically comparably fast as each run of a binary one-vs.-rest SVM. Running 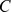 one-vs.-rest binary SVMs for the multi-class problem will therefore require solving 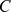 times as many optimization problems of similar computational complexity as Scatter SVM. This may be unfortunate when running times are high and cross-validation is needed. Similar comments also apply to the one-vs.-one approach, requiring 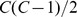 runs, although solving smaller sub-problems at each step. We also develop a chunking-based optimization procedure.

However, although we motivate and develop Scatter SVM by analyzing and extending the dual of 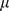-SVMs, the theory is not complete without the corresponding primal. In particular, the score function to be used in the actual classification of test patterns is not revealed by the dual view. Therefore, as a further contribution, we develop the full regularized risk minimization primal of Scatter SVM. The reference to the mean of the prototypes in the dual view translates into a reference to a mean hypothesis, in the form of a hyperplane, in the primal view. Basically, in the primal, Scatter SVM learns class-wise hyperplanes such that the corresponding class scores better than the mean hypothesis by a margin.

The regularized risk minimization framework shows that our initial formulation of Scatter SVM corresponds to the use of the hinge loss in the computation of the empirical risk. The theory may therefore be further developed by incorporating other loss functions. Furthermore, the primal shows how a hyperplane bias parameter affects the constraints in the dual, hence providing additional theoretical insight.

We investigate properties of the new algorithm with respect to speed and sparsity, and report experimental results, which shows that our method may obtain promising performance when compared to the state-of-the-art, at a reasonable computational complexity.

Another interesting subject we explore is the creation of Scatter SVM sensitivity maps. In [20], visualization of the sensitivity of binary SVM solutions to the features (corresponding to brain regions in that work, since the focus was on neuroimaging), both in the linear and the non-linear case through kernels, was enabled through so-called sensitivity maps. In the multi-class case, using e.g. multiple binary SVM classifiers, it is not obvious how to create sensitivity maps. We show that Scatter SVM is well suited to create sensitivity maps also in the multi-class case.

The method [8], which came to our attention in the final phases of this work, leads to an optimization problem which is quite similar to ours, however, from a completely different starting point of convex hulls. Our work surpasses [8] in several aspects. First, by offering a complete dual-primal view, which reveals a bias parameter controlling constraints in the dual and which provides the score function to be used in testing. Our test rule performs better in experiments than the heuristically obtained rule in [8]. Second, by developing a dedicated and fast solver. Third, by discussing different loss functions and the creation of sensitivity maps, which opens up further possibilities within this framework.

## Methods and Theory

We start by reinterpreting the binary SVM method in terms of scatter between class prototypes and their mean. Thereafter, we extend this novel Scatter SVM theory to multiple classes. Furthermore, we examine in detail regularization and loss function issues. Finally, we provide a fast and dedicated solver for the resulting optimization problem.

### A 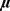-SVM Geometrical Prototype Analysis

In this section, we reinterpret the 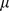-SVM [10] to provide a new geometrical analysis of this classifier in terms of minimization of between-class scatter with respect to class prototypes and their mean. This provides the groundwork for a subsequent multi-class extension.

SVMs are normally defined in terms of a class-separating score function, or hyperplane
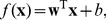
(1)which is determined in such a way that the margin of the hyperplane is maximized. Let a labeled sample be given by 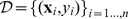, where each example 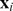 is drawn from a domain 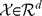 and 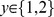. Consider the following optimization problem of training a 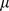-SVM [10]

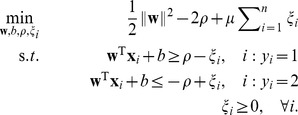
(2)The hyperplane determined by 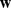 and 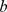 has a functional margin 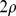 which is explicitly maximized while at the same time obeying the constraints. This is achieved under the regularization imposed by minimizing 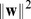. The parameter 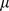 controls the emphasis on the minimization of margin violations, quantified by the slack variables 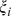.

By introducing Lagrange multipliers 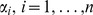, collected in the 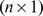 vector 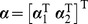, where 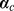 stores 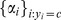, 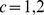, the *dual* optimization problem becomes
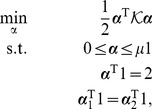
(3)where 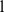 is an all ones vector (the length of 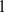 is given by the context) and
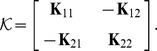
The subscripts indicate the two classes and 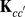 are inner-product matrices within and between classes. Obviously, the constraints in Eq. (3) enforce 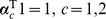.

The optimization determines 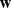 explicitly as
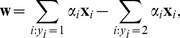
(4)where the non-zero 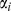
's correspond to the support vectors. The bias 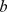 is implicitly determined via the Karush-Kuhn-Tucker (KKT) conditions.

Before proceeding, we note that a formulation of the 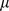-SVM problem with 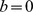 is also possible, corresponding to a score function simply formulated as 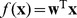, hence requiring that the hyperplane contains the origin. The only change in the dual problem is that the constraint 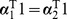 in Eq. (3) disappears. This is a mild restriction for high dimensional spaces, since it amounts to reducing the number of degrees of freedom by one (see also [21]).

The dual formulation of the 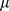-SVM optimization problem has an interesting interpretation in terms of distances between convex hulls, see for example [10], [22]. Here, we provide a new geometrical interpretation in terms of *class prototypes* and the arithmetic *mean* of those prototypes.

**Figure 1:**
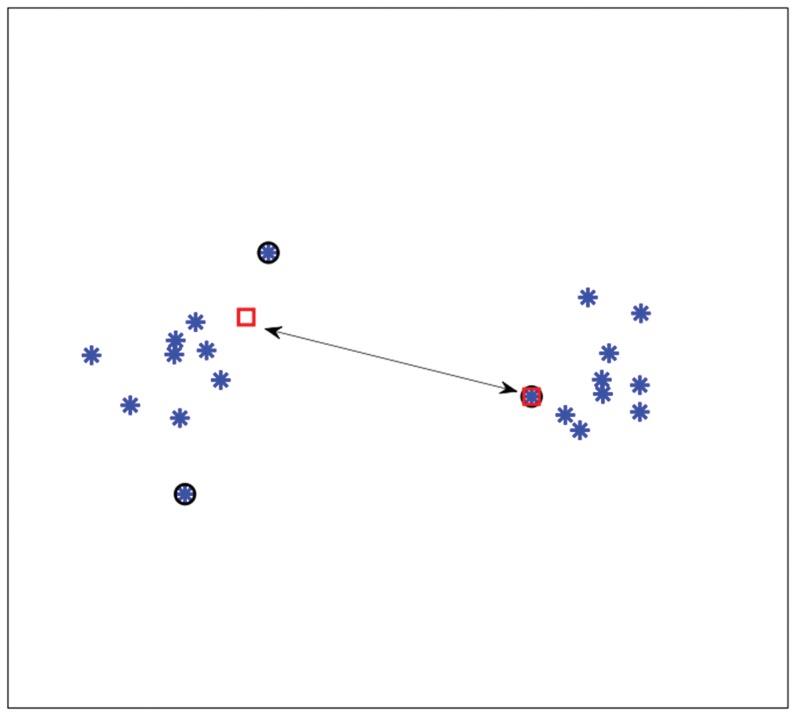
Interpretation of the dual of a 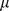
 **-SVM on toy data.**

A versatile way of expressing a prototype 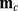 for class 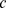 is exemplified by a weighted combination of the data points belonging to that class. Under such a model, the weights determine the properties of the prototype, and thus the way the prototype represents the class. Let
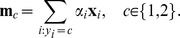
This is therefore an example of a class prototype. Having introduced this notation, the 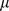-SVM hyperplane weight vector given by Eq. (4) and expressed in terms of prototypes becomes 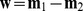. More interestingly, since it is easily shown that 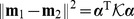, we may by Eq. (3) conclude that the 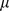-SVM in the *dual* corresponds to minimizing the squared Euclidean distance between the class prototypes 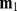 and 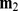. Hence, the optimal class prototypes will be situated on the border between the classes, and not at centers of mass. This is illustrated in Fig. 1. A 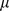-SVM is trained, and the support vectors are shown as the encircled points. The resulting class prototypes are shown as the squares, learned in such a way that the squared Euclidean distance, indicated by the arrow, between these two points is minimized. Situating the class prototypes at the border will “tune” the decision boundary to the border region, which is exactly where the difficult cases to classify are located. This is akin to the minimization of the distance between the convex hulls of the classes [4], [5], also focusing the decision boundary to the border region.

In terms of the class prototypes, the score function, Eq. (1), which is the end product to be used in the testing phase of the classifier, is expressed as 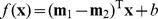 if the bias is included in the primal, or just 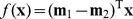 in the case that the bias parameter is omitted from the primal formulation. Very recently, [11] also analyzed several *binary* classifiers and provided related types of prototype frameworks.

We provide a novel addition to the prototype framework by adding the arithmetic mean 
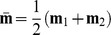
 into the picture. In terms of the mean and the class prototypes, we may rewrite Eq. (3) to obtain the equivalent expression
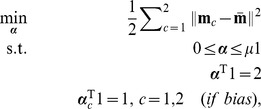
(5)since 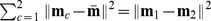 up to a constant. Interestingly, this new geometrical way of viewing the dual of the 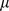-SVM may be related to the notion of *scatter* in pattern recognition.

The so-called between-class scatter is normally defined as 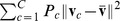 [23], [24], with respect to class means 
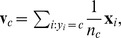
 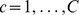, and the global mean 
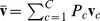
. The prior class probability is 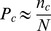 where 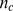 is the cardinality of the 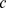
'th class. Hence, for 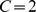, by introducing the weights 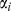 for each data point 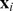 and by defining the scatter with respect to the class prototypes 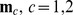, and their arithmetic mean under the equal class probability assumption, the cost function 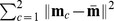 is obtained.

If the score function 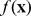 includes the bias parameter 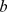, we see that the 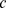
'th class prototype is restricted to the convex set defined by the members of that class since the class-wise weights sum up to one. If the bias is omitted, the class prototype is not limited to the convex set.

**Figure 2:**
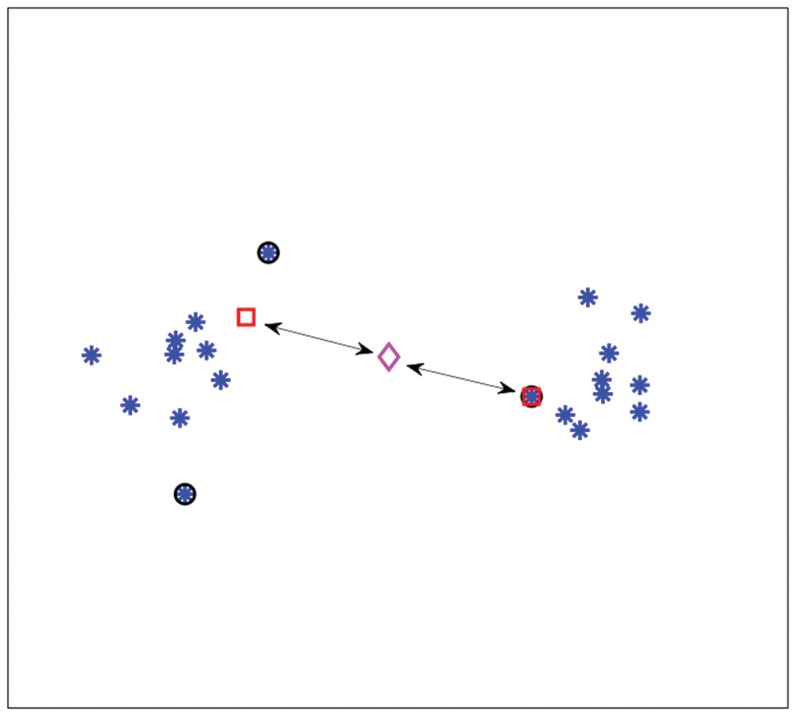
Scatter interpretation of the dual of a 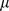
 **-SVM on toy data.**

Fig. 2 shows an interpretation of the 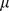-SVM in terms of scatter. The arrows indicate the squared Euclidean distance between the class prototypes and their mean (“diamond”). The sum of these distances, i.e. the scatter, is minimized.

The prototype and scatter-based viewpoint of the 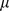-SVM training phase introduced here not only provides a new interpretation. The practical benefit is that it suggests a computationally efficient extension to multiple classes, since scatter is inherently a multi-class quantity. This topic we explore in the next section. An incorporation of unequal class priors may also be worthwhile a study, but will not be pursued here.

### Scatter SVM: A Multi-Class Extension

In this section, we motivate and analyze an algorithm for *training* a joint, or multi-class, 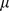-SVM-inspired learning machine, by extending the scatter-based view of the dual of the binary 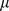-SVM to multiple classes. Aspects which concern the actual score function to use in testing, with and without bias, is deferred to the next section, where we derive the full regularized risk minimization framework.

By Eq. (5), a direct extension of the scatter-based view of the dual to 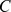 classes is proposed here as
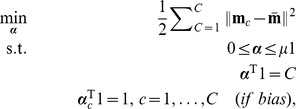
(6)for 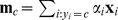, 
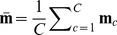
 and weights 
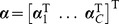
, where 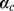 stores 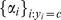, 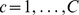. This constitutes a direct extension of scatter to multiple classes. In this formulation, it is optional whether or not to include the last constraint, depending on a bias parameter discussed shortly.

It is easily shown that 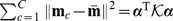, up to a constant, where
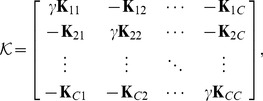
(7)

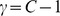
 and 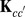 are inner-product matrices within and between classes. Hence, the optimization problem Eq. (6) may also be expressed as
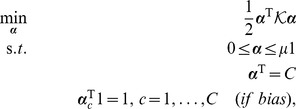
(8)The matrix 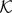 is 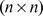 and positive semi-definite, and therefore leads to an optimization problem over a quadratic form (cf. Eq. (8)), which constitutes a convex cost function. The box constraints enforce 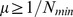 where 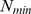 is the number of points in the smallest class. This problem can be solved efficiently by quadratic programming. There are merely 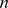 variables to be optimized, as opposed to 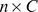 variables for joint approaches like [13], [14]. With the bias included, there are 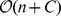 simple constraints. This problem is basically equal to [8]. However, if the bias is omitted, there are even less constraints, only 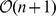. This latter optimization problem is the one we primarily focus on in the experiments. We are thus faced with an optimization problem of much lower computational complexity than previous joint approaches. In fact, our optimization problem Eq. (8) lends itself nicely to a solver based on sequential minimal optimization [25] or chunking optimization [26], respectively, depending on whether the bias is included or not. We discuss this shortly, providing a computationally efficient and fast Scatter SVM algorithm.

Note that in [8], Matlab's *quadprog* solver was used, which according to the authors of that paper, “takes no advantage of the structure of the problem”. Furthermore, they state that “the design of a fast iterative solver … is a key research challenge…”.

**Figure 3:**
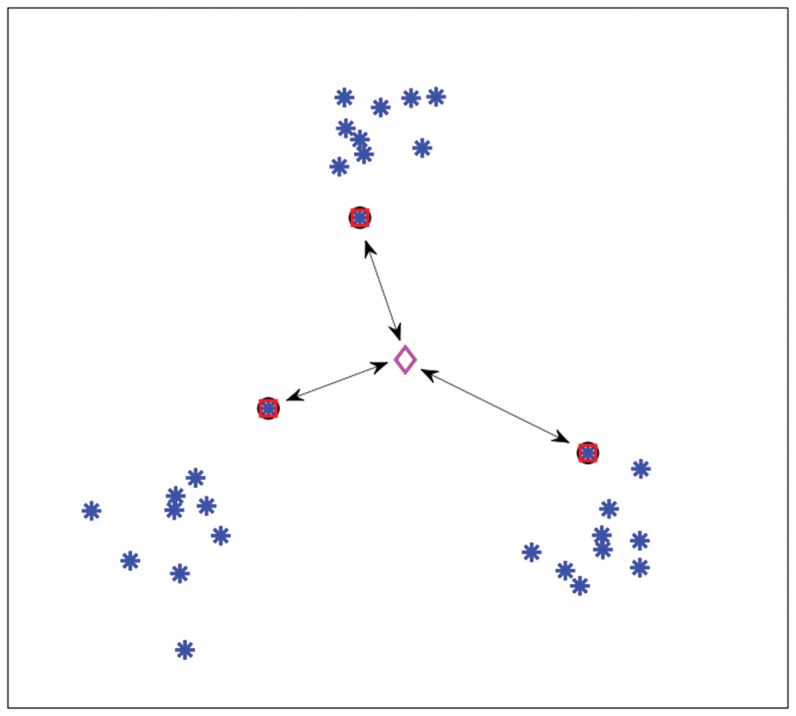
The result of training Scatter SVM on three classes (toy data set).

Figure 3 shows a simple three-class example of training the Scatter SVM. Again, the encircled points show support vectors, and squares show the class prototypes. In this example, there is only one support vector for each class, and consequently the class prototypes equal the support vectors. The arrows indicate the squared Euclidean distance between prototypes and their mean. The sum of these distances, i.e the scatter, is minimized by the training procedure. Note that two of the classes are equal to the two-class data set shown in Fig. 2. The support vectors for these two classes are no longer the same in Fig. 3 as compared to Fig. 2. This is perfectly logical, as the two classes are now part of a larger joint optimization problem.

**Figure 4:**
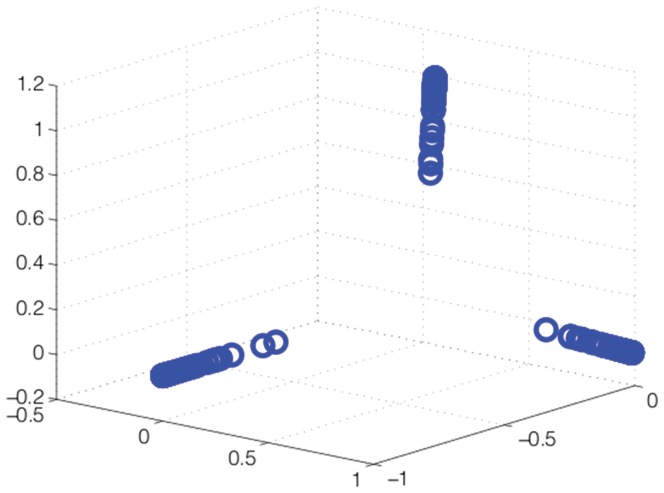
Illustration of a kernel induced mapping of data.

Of course the toy data set in Figure 3 has a “benign” structure for Scatter SVM training, in that the classes are nicely distributed around a center point. It is obvious that one may construct cases, for example several classes distributed along a line, where the reference to the mean of the class prototypes may be problematic. However, by mapping the data to a richer space, of higher dimensionality, such issues are avoided. A simple example may illustrate this point. A one-dimensional data set is created, with three well-separated classes having class means at 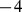, 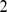 and 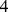, respectively, and global mean equal to 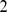. With the use of a Gaussian kernel, this data set is mapped into a high dimensional space. For visualization, we show in Fig. 4 the corresponding empirical kernel PCA [2], [27], [28] map obtained using the three largest eigenvalues. This yields a three-dimensional space in which the classes are nicely distributed wrt. the mean.

For this reason, and also for increasing the probability of linearly separable classes by Cover's theorem [29], we in general employ the kernel induced non-linear mapping
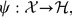
(9)to a Hilbert space 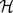 [30]. Kernel functions 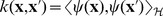 are thus utilized to compute inner products in 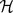.

We have proposed to extend the dual of the 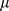-SVM to multiple classes, and have reached an optimization problem which is manageable in the number of variables and constraints. However, we have yet to explain the rationale behind the last optional constraint in Eq. (6) and Eq. (8), and we have also not discussed the actual score function to use in the testing phase of Scatter SVM. As explained in the previous section, in the two-class case, it is the primal formulation of the problem in terms of a score function that may or may not include a bias term, that determines whether or not to include the last constraint in Eq. (5). Hence, in order to obtain a consistent theory, we need to derive the primal problem leading to the dual which constitutes Scatter SVM. This is the topic of the next section.

### Regularization Framework, Loss and Prototype Score Function

In this section, with the accompanying detailed derivations in Appendix S1, we provide the primal view of the Scatter SVM in a full regularized risk optimization framework, which reveals the score function to be used to classify unseen data points. Fenchel-Legendre dualization reveals the form of the dual optimization problem, and involves loss functions which opens up further possibilities for Scatter SVM learning. The primal derived here incorporates the primal of the binary 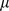-SVM as a special case.

Let the goal be to find a hypothesis 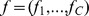 that has low error on new and unseen data, where the *scoring functions* 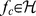. Labels are predicted according to
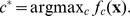
Applying regularized risk minimization returns the minimizer 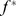, given by
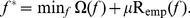
(10)The regularizing function is determined by 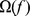 and the empirical risk of hypothesis 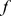 is given by
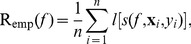
(11)with respect to a convex loss function 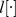. In the remainder, we focus on affine-linear hyperplane models of the form
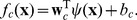
(12)As discussed earlier, the bias parameter 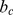 may be removed in the derivations, which is a mild restriction for the high dimensional space 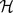 we consider.

**Figure 5:**
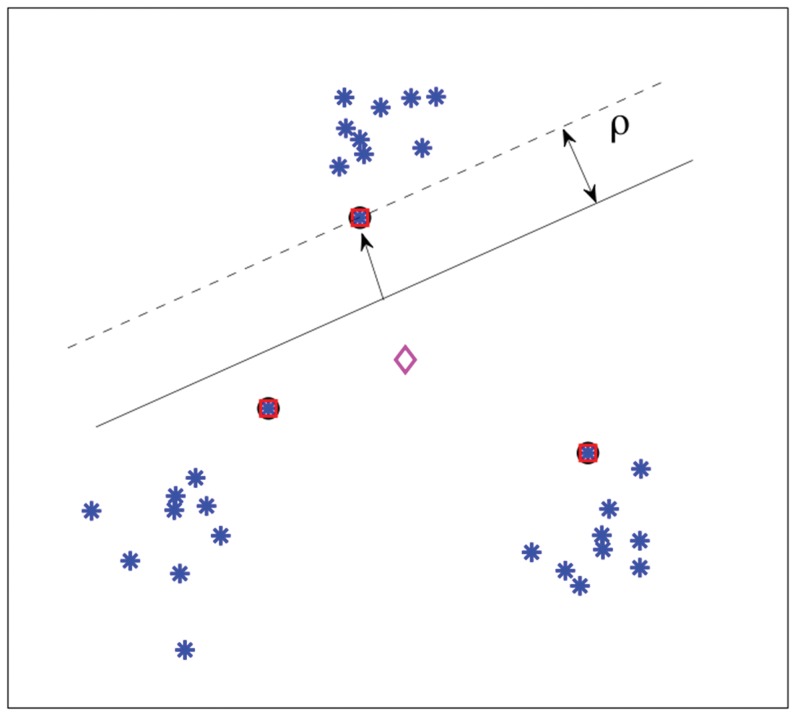
In the primal, Scatter SVM learns class-wise hyperplane functions. The function for class 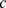 scores better than the average hypothesis by a margin, for all class 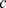 training data points.

A key quantity is 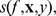 the argument of the loss function. As a novel contribution, we propose to compute loss based on a comparison between the performance of hypothesis 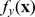 and the *average* hypothesis (hyperplane) 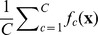, by
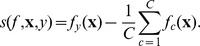
Expanding the loss terms into slack variables 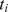 leads to the *primal optimization problem* (see Appendix S1)
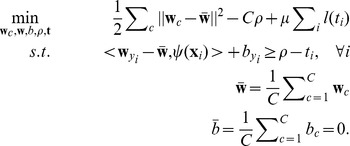
(13)Here, 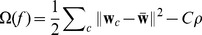. Note that the constraint 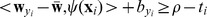 implies that the class-wise hyperplane for class 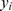 scores better than the average hypothesis by a *margin*. See Fig. 5 for an illustration.

By Fenchel-Legendre dualization, we obtain 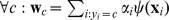, yielding the generalized dual problem
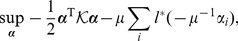
(14)where 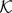 is given by Eq. (7), 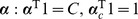, 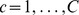 (if bias) and where 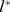 is the *dual loss* of 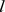 [31].

#### Hinge Loss Dual Yields Scatter SVM

When utilizing the *hinge loss* 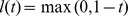 into Eq. (14), noting that the dual loss is 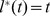 if 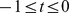 and 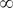 elsewise (cf. Table 3 in [31]), we obtain the dual
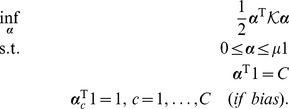
(15)where 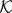 is given by Eq. (7). Obviously, the dual equals Eq. (8) and is thus equivalent to the *scatter minimization procedure* discussed in Section 0. The last constraint only applies if the bias parameter is included in the score functions, Eq. (12).

#### Alternative Loss Functions

The above analysis shows that Scatter SVM, as derived in this paper, corresponds to the use of the hinge loss. For completeness, we mention that using e.g. *squared hinge loss*, 
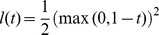
 which gives dual loss 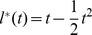 if 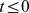 and 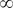 elsewise, produces a dual problem
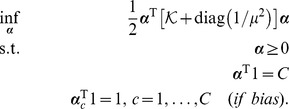
(16)The difference to the former problem lies in the increased conditioning term diag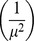 of the kernel matrix and in that no upper bounds on 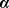 are imposed.

Another interesting loss function is *logistic loss*. In that case, 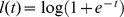 which gives dual loss 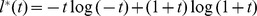 if 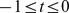 and 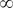 elsewise, producing the dual
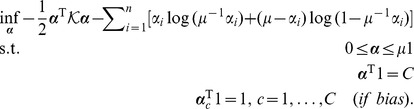
(17)


It is left to future work to investigate these formulations, or other formulations based on different losses, but it illustrates some of the versatility of our approach with respect to the loss function.

#### Prototype Score Function in Dual View

As mentioned above, by dualization, the hyperplane weight vector is given by 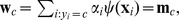 i.e. a class prototype by our previous notation. The labels are therefore predicted according to
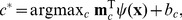
(18)if the bias is included in the primal, or simple as 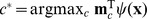 if the bias is omitted from the primal.

### A Fast and Dedicated Shogun Implementation

With ever increasing data sets, solving complex mathematical programs such as the one in Eq. (15) with off-the-shelf solvers quickly becomes impractical. Here we describe an implementation of a dedicated efficient high-performance solver for the Scatter SVM quadratic program, that emerges from binary SVM solvers.

Many efficient SVM training methods rely on decomposition techniques; examples are chunking [26] and SMO. The idea of decomposition is to iteratively improve a solution candidate by solving a sequence of subproblems: to optimize a small number of variables (the so-called working set) while, for that moment, freezing all others. In chunking, the subproblems typically contain a few dozen variables and may be solved with off-the-shelf optimizers. In SMO, the working sets consist of exactly two variables, such that analytical optimization is possible.

Apart from the working set size, the critical design choice is the selection of the variables for the sub-problem: the convergence speed for the global optimization depends on the amount of progress that the sub-problems allow for. To make SMO efficient, clever selection strategies for the two variables 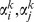 to be optimized at iteration 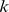 are required. A proven strategy based on second order information is implemented in LIBSVM [32], [33]. In chunking-based optimization the subset selection is less critical since a block of variables is active at the same time increasing the chance of having a good set.

We exploit the fact that our problem Eq. (15) is a close relative of the 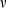-SVM dual in the with-bias case and of the C-SVM in the without-bias case. However, LIBSVM is only capable of a with-bias training. We thus implemented two algorithms in the SHOGUN toolbox [34]: a SMO implementation of 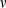-LIBSVM for the with-bias training and a chunking implementation of SVMlight for the without-bias training. We refer to Appendix S2 for more details on the implementation.

Both versions are publicly available for download at http://www.shogun-toolbox.org/.

## Experiments

The aim of the experimental section is to highlight properties of Scatter SVM in terms of sparsity, generalization ability and computational efficiency, by performing classification on some well-known benchmark data sets used in the literature (see e.g. [16], [35]). Furthermore, as a novel addition to the multi-class support vector machine literature, we also develop sensitivity maps for illustrating the relative importance of the underlying features to the classification result obtained by Scatter SVM.

In all experiments, the RBF-kernel is adopted. This is the most widely used kernel function, given by
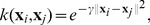
(19)where 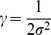.

### Experiment on Controlled Artificial Data

**Figure 6:**
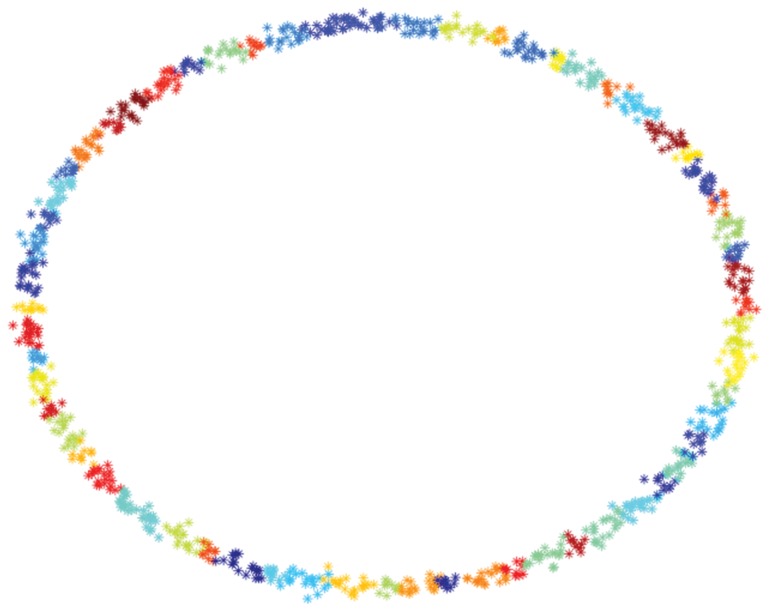
Visualization of toy data sets: 100 class circle data set.

**Figure 7:**
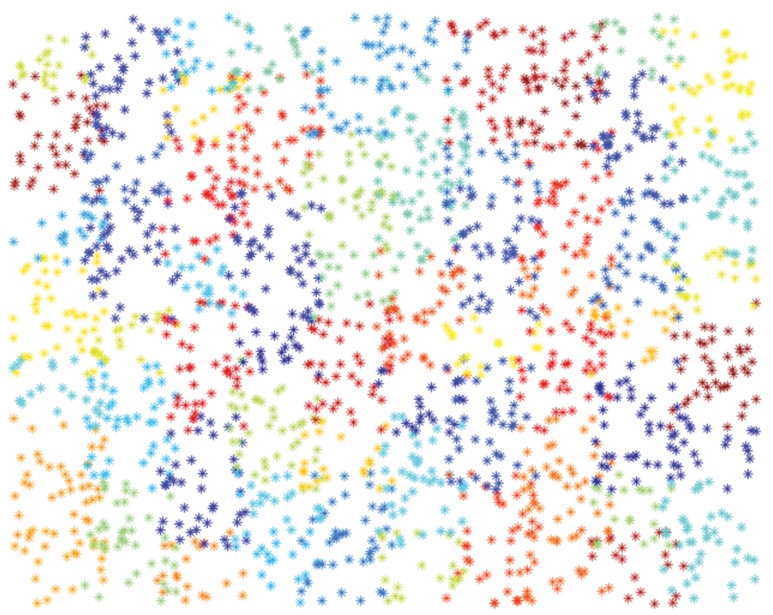
Visualization of toy data sets: 100 class checker data set.

As a first experiment, we perform a “sanity” check of the Scatter SVM algorithm in a controlled scenario, focusing both on computational efficiency and generalization ability. To this aim, we artificially generated two data sets that have been often used in the literature (e.g. see [30]): 2d-checker-boards with slightly overlapping fields and one class for each field, and 2d-data sets of Gaussians evenly distributed on a circle. These data sets are illustrated in Fig. 6 and Fig. 7.

Both the number of classes and the number of data points are increased (cf. Table 1). For the checker (circle) data set we generated 20 (10) points per class and split the data set evenly into training and validation set (with an equal number of points in each class). For this experiment, the Scatter SVM is executed in with-bias mode, and is contrasted to a one-vs.-rest (OVR) C-SVM (more thorough experiments with comparisons to other SVM approaches are deferred to a later subsection). Both methods are based on LIBSVM as implemented in the SHOGUN toolbox. We perform model selection over the parameters on the validation set. For SVMs, RBF-kernels of width 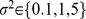, 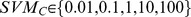, and 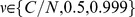 are used. We then measure time (training+prediction) and classification error rates (in percent, rounded) for the *best* performing model.

**Table 1:** Time comparison of the proposed Scatter SVM to the OVR LIBSVM training strategy.

		Checker-Board			Circle			
Error [%]	OVR SVM	35	49	50	22	24	22	21
Error [%]	Scatter SVM	24	40	41	14	17	18	17
Time (s)	OVR SVM	0.05	1.77	102.15	0.02	3.51	1,229.30	197,236.71
Time (s)	Scatter SVM	0.06	1.59	85.21	0.01	2.11	46.27	42,401.26
#Classes		10	100	1,000	10	100	1,000	10,000
N		200	2,000	20,000	100	1,000	10,000	100,000

With reference to Table 1, the execution times of Scatter SVM compare favorably to the OVR C-SVM, and in the most extreme case correspond to a speed up factor up to 27. Scatter SVM achieves a higher generalization ability than OVR. This might be because these data sets contain a fixed number of examples per class and are thus well suited for Scatter SVM. In other words, selecting this data may imply a bias towards Scatter SVM. However, these experiments illustrate in particular the speed-up properties of our algorithm while maintaining good generalization.

### Case-Based Analysis of Support Vectors and Sparsity

We perform two experiments in order to analyze the support vectors created by Scatter SVM, focussing on handwritten digits since such data is ideal for visualization. A three-class data set is created by extracting the classes corresponding to the digits “0”, “6” and “9” from the U.S. Postal Service (USPS) data set [36]. We randomly select 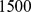 data points for training, and use 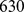 data points as a validation set to determine the kernel size for which to display the support vectors. A five-class data set is also created by randomly extracting 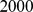 data points representing classes “0”–“4” in the MNIST data set [37], randomly separated into a training set and a validation set. For MNIST, all data points are normalized to unit length.

For this, and all remaining experiments, Scatter SVM operates in the without-bias mode, based on a SHOGUN SVMlight implementation. Note that in pre-experiments we compared the with- and without-bias training and found that the without-bias Scatter SVM, involving the least number of constraints, consistently achieves either an equal or slightly better test error. We thus only present results for the without-bias case and refer the reader interested in the with-bias results to our previous technical report [38]. In that report, results were also contrasted to the method in [8]. The training procedure in [8] resembles our with-bias training, but the testing rule is based on heuristics. It did not perform satisfactorily in our experiments, therefore these results are not repeated here. The “
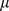
” parameter in Scatter SVM translates into a “
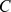
” parameter, similar to the parameter in the OVR C-SVM. Both methods are now trained on eleven logarithmically evenly spaced 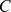-parameters from 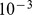 to 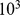. The validation procedure is performed over 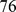 kernel sizes 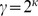 for 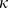 between 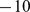 and 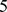 in steps of 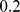 in Eq. (19).

**Figure 8:**
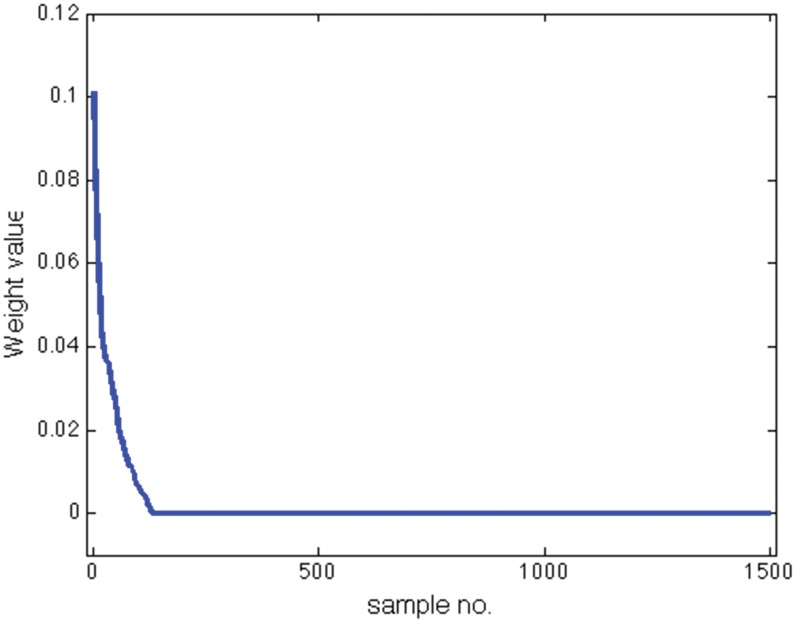
Analysis of Scatter SVM sparsity on USPS data.

**Figure 9:**
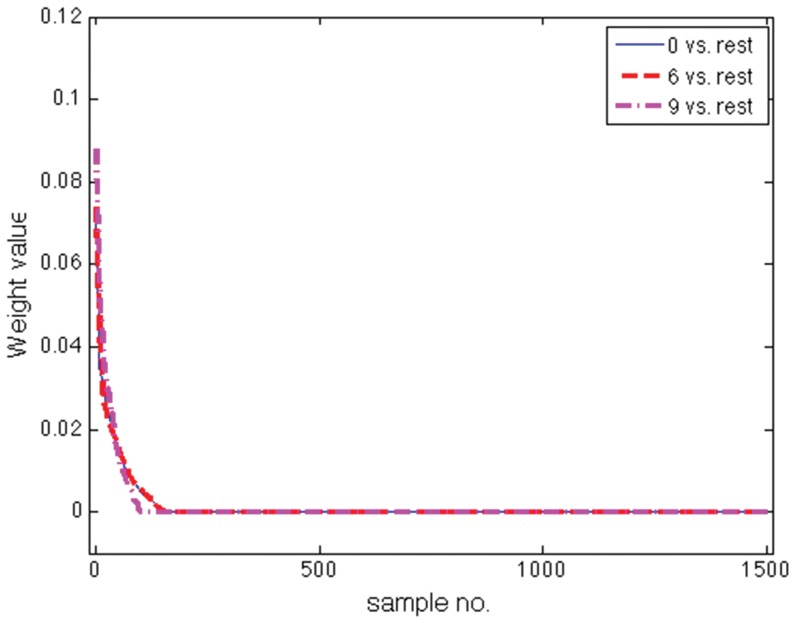
Analysis of OVR SVM sparsity on USPS data.

On the current three-class USPS data, Scatter SVM and the OVR C-SVM obtain best validation results corresponding to 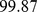 and 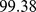 percent success rate, respectively. Figure 8 shows the weights 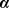 obtained by Scatter SVM sorted in decreasing order. A majority of the weights are zero. In Fig. 9, the three sorted weight vectors obtained by the OVR C-SVM is shown, also demonstrating a certain sparseness.

If we define a 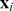 as a support vector if 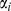 is greater in value than 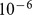, then Scatter SVM produces 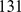 SVs, corresponding to 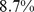 of the training data. The number of SVs for each class is shown in Table 2, together with the SV structure for the C-SVM. The number in parenthesis indicate the number of unique SVs of that class obtained in the “rest” part of the training. The number of all unique SVs is 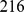 corresponding to 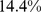 of the training data.

**Table 2:** USPS-based analysis of support vector sparsity.

USPS # SVs	0	6	9
Scatter SVM	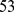	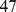	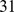
OVR SVM	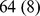	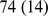	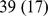

**Figure 10:**
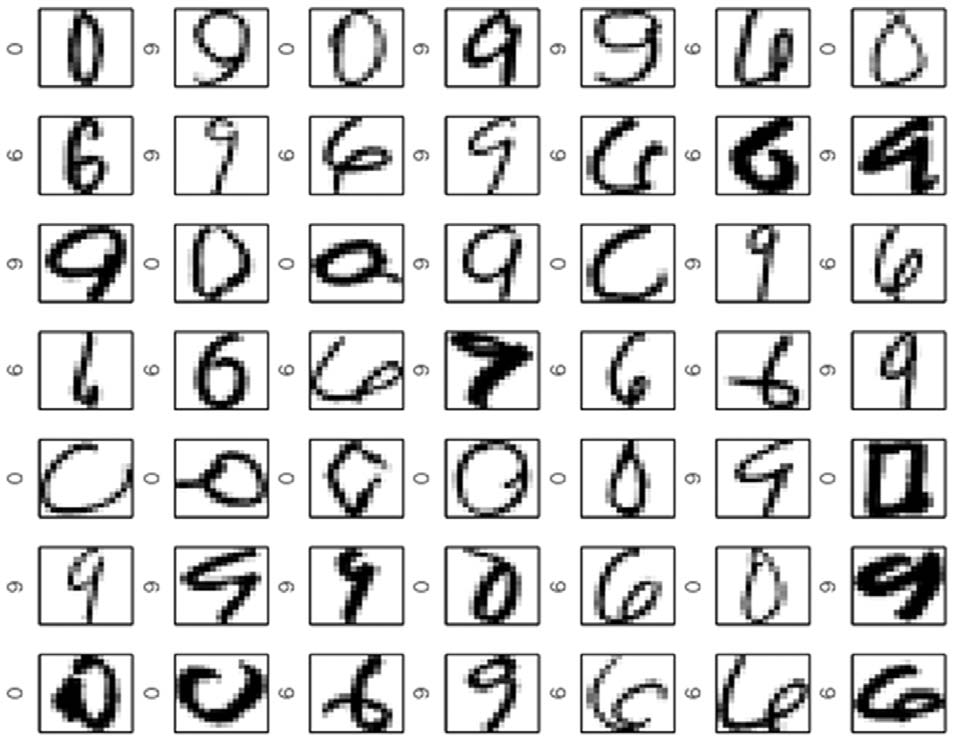
Scatter SVM support vectors on USPS data.

**Figure 11:**
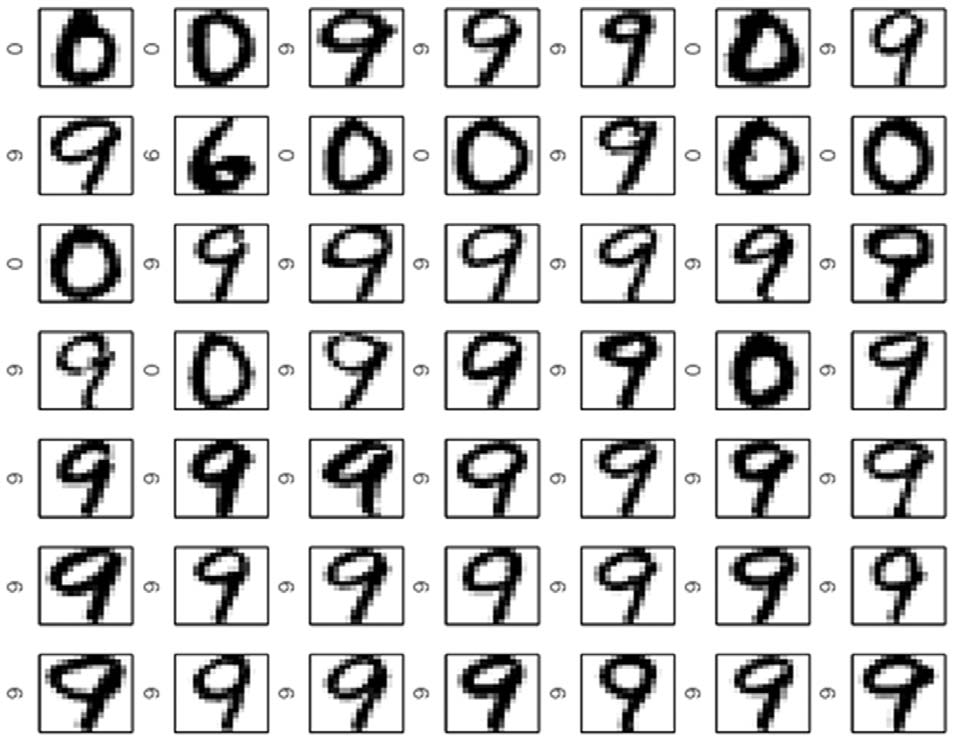
Scatter SVM non-support vectors on USPS data.

Figure 10 shows the largest 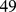 support vectors in decreasing order from upper left to bottom right wrt. the weights. Labels are indicated. In a manner common to other support vector-based methods, the Scatter SVM SVs represent the “difficult” data points to classify. In contrast, Fig. 11 shows the digits corresponding to the smallest weights (all basically zero). These are all neatly written.

A similar experiment is performed for the MNIST data, here with five classes. Scatter SVM obtains a best validation result of 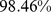, while the C-SVM's best result is 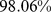. Table 3 summarizes the number of SVs. There are 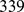 Scatter SVM SVs, corresponding to 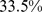 of the training data. The C-SVM produces 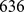 unique SVs, i.e. using 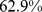 of the training data.

**Table 3:** MNIST-based analysis of support vector sparsity.

MNIST # SVs	0	1	2	3	4
Scatter SVM	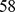	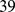	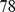	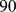	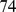
OVR SVM	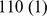	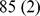	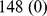	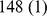	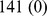

**Figure 12:**
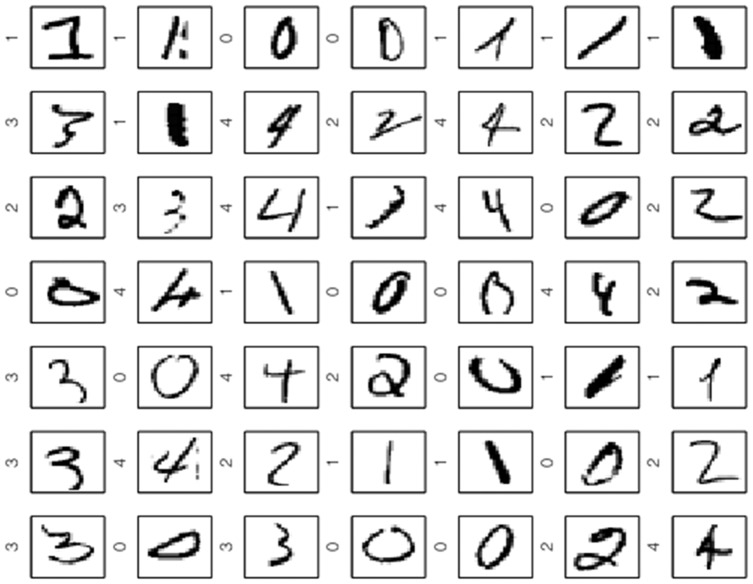
Scatter SVM support vectors on MNIST data.

**Figure 13:**
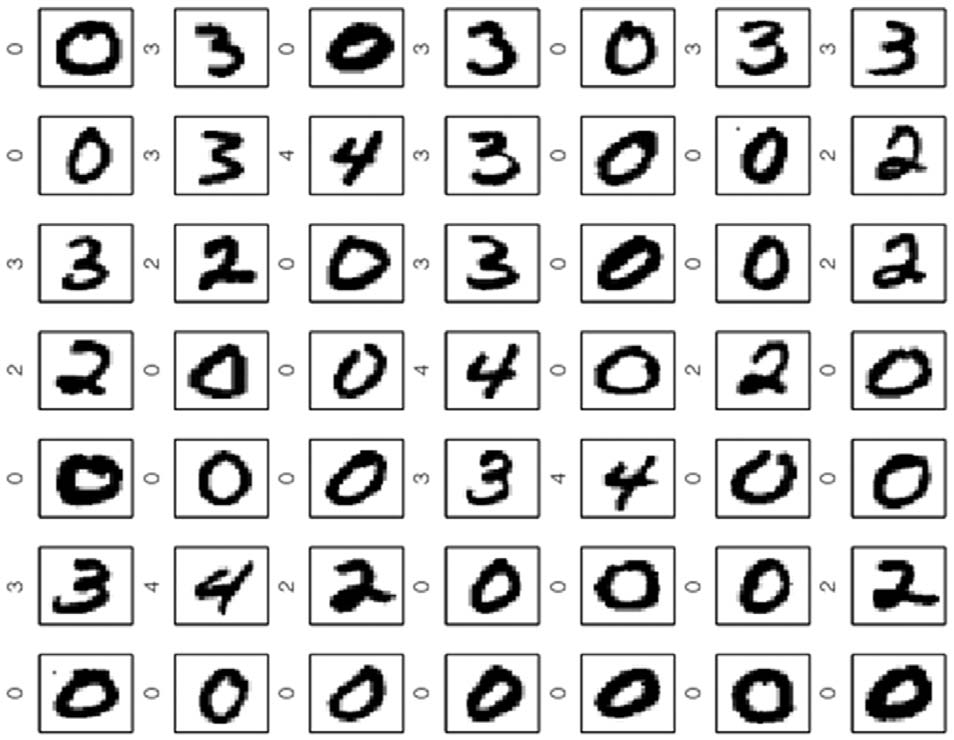
Scatter SVM non-support vectors on MNIST data.

Figure 12 and Fig. 13 shows the largest Scatter SVM SVs and the smallest non-SVs, respectively. Also in this case, the SVs display a wide variety of shapes within classes, as opposed to the non-SVs.

These experiments show that Scatter SVM may perform on par with or better than an OVR C-SVM with respect to the sparsity of the solution. This we consider encouraging.

### Generalization Ability on Benchmark Data Sets

To investigate further the generalization ability of Scatter SVM, we perform classification experiments on some well-known benchmark multi-class data sets commonly encountered in the literature (see e.g. [13], [16], [35]). The data sets are listed in Table 4. For those cases where specific test data sets are missing, we perform 10-fold cross-validation over the parameters and report the best result. If a test set is available, we simply report the best result over all combinations of parameters. In a practical situation, a validation set would of course be used to determine the appropriate parameters. The data sets are obtained from the LIBSVM web-site: http://www.csie.ntu.edu.tw/~cjlin/libsvmtools/datasets/multiclass.html, (except MNIST) pre-processed such that all attributes are in the range 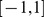. The MNIST data, obtained from http://cs.nyu.edu/~roweis/data.html, is normalized to unit length.

**Table 4:** Multi-class benchmark data sets used in generalization study.

	# training data	# testing data	# class	# attributes
Iris	150		3	4
Wine	178		3	13
Glass	214		6	13
Vowel	528		11	10
Segment	2310		7	19
MNIST (0–4)	2000		5	784
Satimage	4435	2000	6	36
Dna	2000	1186	3	180
USPS	7291	2007	10	256

In this experiment, the Scatter SVM is contrasted to OVR C-SVM, one-vs.-one (OVO) C-SVM and Crammer and Singer's (CS) [16] multi-class SVM since these are related prototype-based methods. Crammer and Singer's method constructs as many hyperplanes as there are classes, each based on 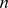 variables. Hence, the optimization problem may be quite large. Compared to methods like [13], [14], the approach is much simpler since there are only 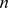 slack variables 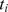, obtained by constructing a scoring function based on the maximum gap among all the hyperplanes and the 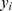
'th hyperplane. Decomposition methods were proposed in order to reduce computational complexity. Still, the method is vastly more complex than OVR or OVO approaches. All methods are trained for the same set of parameters and kernel sizes as in the previous section. For completeness, we also compare with a Naïve Bayes (NB) classifier based on kernel density estimation [39]. The best NB result over the same range of kernel sizes used in the SVM methods is shown in each case.

The results, shown in Table 5, indicate that Scatter SVM has been able to generalize well, and to obtain classification results which are comparable to these state-of-the-art alternatives. Considering that Scatter SVM constitutes a more restricted model with far less variables of optimization, we consider these results encouraging, in the sense that Scatter SVM may perform well at a reduced computational cost. For example, running CS on the “Vowel” data (full cross-validation) required 3 days of computations. All the three other methods only required a small fraction of that time.

**Table 5:** Classification results on several real-world data sets.

	Scatter SVM	OVR SVM	OVO SVM	CS	NB
Iris	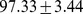	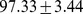	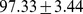	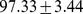	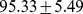
Wine	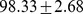	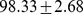	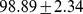	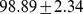	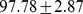
Glass	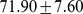	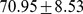	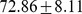	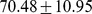	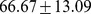
Vowel	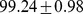	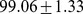	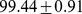	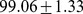	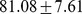
Segment	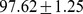	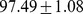	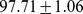	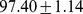	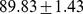
MNIST (0–4)	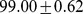	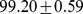	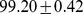	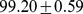	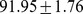
Satimage	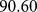	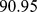	91.00	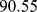	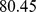
Dna	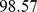	98.40	98.31	98.31	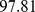
USPS	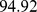	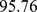	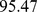	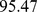	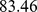

The tendency seems to be that where the results differ somewhat, the OVO C-SVM, in particular, has an edge. This is not surprising compared to Scatter SVM, since the reference to the global mean in Scatter SVM introduces a form of stiffness in terms of the regularization of the model, which will require a certain homogeneity among the classes, with respect to e.g. noise and outliers, to be at its most effective. For noisy data sets, a more fine grained class wise regularization approach will have many more variables of optimization available to capture the fine structure in the data, at the expense of computational simplicity. The USPS data may represent such an example, where Scatter SVM performs worse than all the alternatives.

Even though the primary focus of this paper is performance and computational efficiency in multi-category classification, we include some remarks about the high dimension, low sample size (HDLSS) scenario, which has received recent interest, e.g. in microarray data analysis. In [40], it was shown that HDLSS data tends to lie deterministically at the vertices of a regular simplex, and that different classification methods operate differently under such conditions. In order to briefly investigate Scatter SVM in the HDLSS setting, firstly, we reduce the MNIST (0–4) data set by a factor 6, such that each class contains on average about 65 samples (334 points in total), each with a dimension of 784. Secondly, we reduce the USPS training set by a factor 20, such that the smallest class contains only 19 points, and the largest class 64 points (364 points in total). The dimension is 256. The classification results obtained are shown in Table 6. Perhaps surprisingly, on MNIST (0–4), the Scatter SVM increases its mean accuracy rate compared to the full data set, although with larger standard deviations. On the USPS data, where Scatter SVM performed worst on the full data set, the gap to the other methods increases. Overall, this brief analysis indicates no clear differences wrt. the alternatives when it comes to performance in the HDLSS setting. A comparison and thorough analysis akin to recent results on distance weighted discrimination methods [41] would be interesting, but is deferred for future work.

**Table 6:** Classification results in the HDLSS setting on MNIST (0–4) and USPS.

HDLSS	Scatter SVM	OVR SVM	OVO SVM	CS
MNIST (0–4)	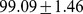	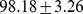	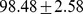	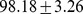
USPS	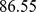	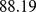	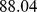	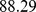

For completeness, we also display the running times of the different algorithms in Table 7 (leaving out the data sets with the lowest sample sizes, where Scatter, OVO and OVR SVMs run basically equally fast). For the data sets where cross-validation is used, we show mean training and testing times over the 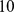 folds and over all kernel widths, which are the same as above. All methods operate with 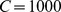. Note that here Scatter SVM is executed in without-bias mode based on a chunking optimization, as opposed to the SMO strategy employed in OVO and OVR SVMs. This influences running times, and the numbers might be different for Scatter SVM optimized using SMO. We note that for USPS, Scatter SVM is the fastest method, and is much faster in general than the CS method, which is also a joint SVM approach. Overall, however, OVO SVM is the fastest method for these data sets. It is a bit surprising that for MNIST, Scatter SVM has a significantly higher training time than e.g. OVR SVM. The Dna data set is binary, and may exhibit an unusual structure compared to the other data sets. Here, the CS method is as fast as Scatter SVM, which is by far not the case on any other data set.

**Table 7:** Running times in seconds (train, test) on several real-world data sets.

	Scatter SVM	OVO SVM	OVR SVM	CS
Segment	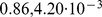	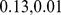	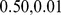	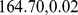
MNIST (0–4)	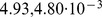	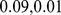	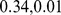	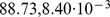
Satimage	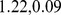	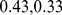	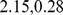	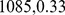
Dna	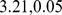	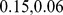	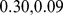	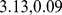
USPS	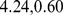	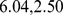	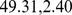	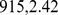

### Computational Efficiency and Speed

In this subsection, in a manner similar to Sec. 0, we would primarily like to indicate the computational efficiency of Scatter SVM, in terms of training and testing times, as a function of the number of classes. To this end, we obtained the Caltech101 data set [42] (from Peter Gehler) including the kernel matrices precisely as used in [43], with the same train/test partition consisting of 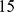 and 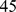 instances per class for training and testing, respectively. We compare the test errors, training time, and testing time attained by Scatter SVM, employing the without-bias implementation, with the ones of OVO, OVR and the implementation of Cramer & Singer. For OVO we used the efficient LIBSVM implementation and for OVR we used SVMlight as binary C-SVM solver.

**Figure 14:**
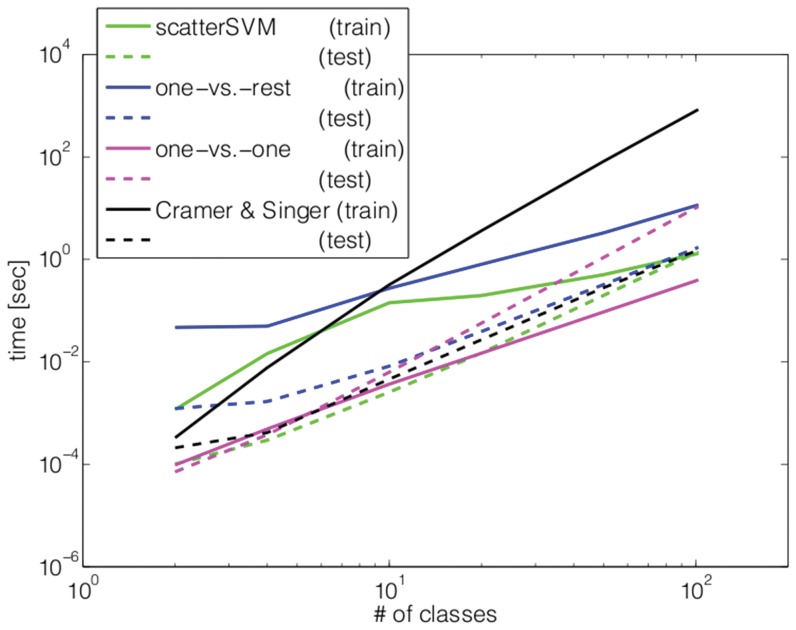
The results of training several SVM-related multi-class methods on the Caltech101 data set in terms of runtime.

Figure 14 shows the training and testing times obtained in this experiment. We can see from the plots that Scatter SVM clearly has the fastest testing time for all numbers of classes, and is also more efficient than OVR and Cramer & Singer in terms of training times. Asymptotically, we observe that with increasing number of classes Scatter SVM scales best in terms of training time so that eventually it will be the fastest method. This asymptotical behavior is already observed in practice: already for 50 classes, Scatter SVM is the fastest method in total (i.e., training time+testing time). We can also remark that Cramer & Singer is by far the slowest method in total due to its exorbitantly high training time.

**Figure 15:**
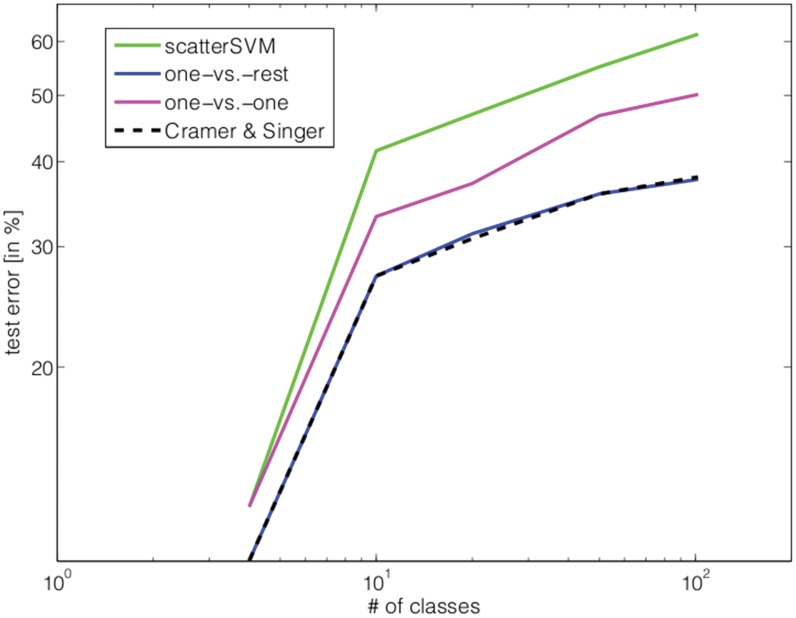
The results of training several SVM-related multi-class methods on the Caltech101 data set in terms of test error.

For completeness, we show in Fig. 15 the observed test errors. The results indicate, on this particular data set, that the Scatter SVM is not able to achieve as low test error rates as the competitors, which are optimizing more variables. This may be due to the very low, and heterogeneous nature, of training samples per class, as discussed in the previous subsection.

### Scatter SVM Sensitivity Maps

In [20], visualization of the sensitivity of binary SVM solutions to the features, both in the linear and the non-linear case through kernels, was enabled through so-called sensitivity maps. In the multi-class case, using e.g. multiple binary SVM classifiers, it is not obvious how to create sensitivity maps. We show that Scatter SVM, on the other hand, is well suited to create sensitivity maps also in the multi-class case.

The sensitivity map (see [20] and references therein) visualizes the relative importance 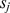 of the input data features 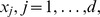 where 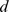 is the dimensionality, for a given function 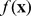 in a stochastic environment with a distribution over the inputs given by the probability density function 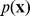, and is given by
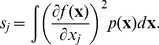
(20)In Scatter SVM, we wish to visualize the sensitivity with respect to the class prototype scoring functions 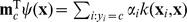 or with respect to the mean function 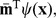 where 
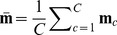
 (note that bias terms are omitted as 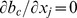). Focusing on class-wise sensitivity using an empirical estimate over 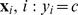, we obtain
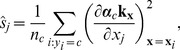
(21)where 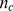 is the cardinality of the sample, 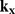 is a 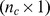 vector that holds elements 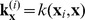. The derivative can be calculated for different types of kernels, including the RBF kernel. See [20] for details. Overall, or mean, sensitivity over all classes is easily computed based on this expression.

**Figure 16:**
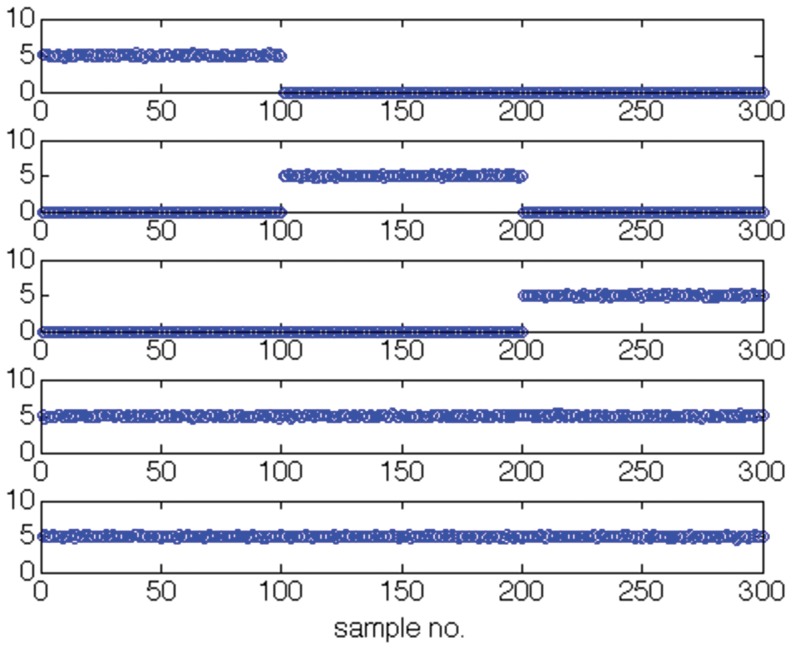
Illustration of toy data used for creating sensitivity plot.

**Figure 17:**
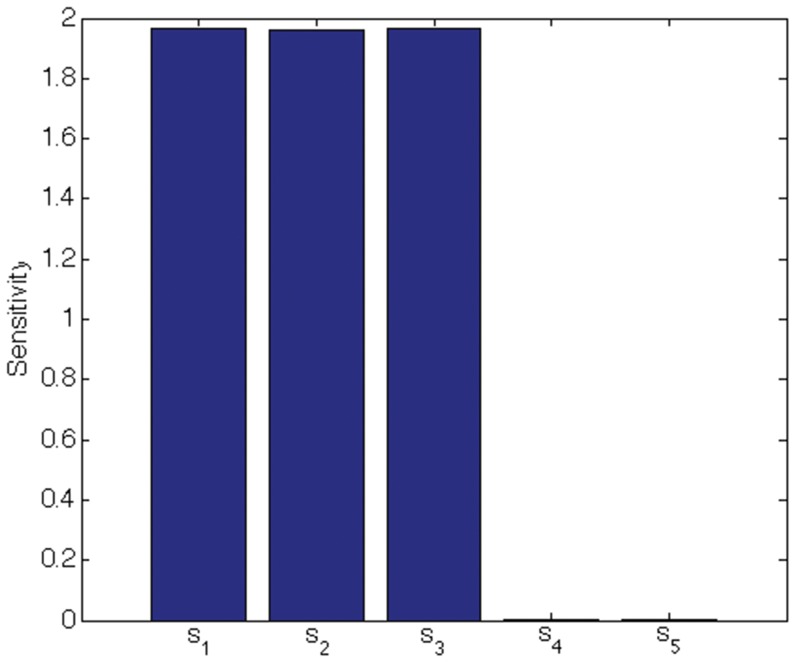
Total sensitivity for toy data.

**Figure 18:**
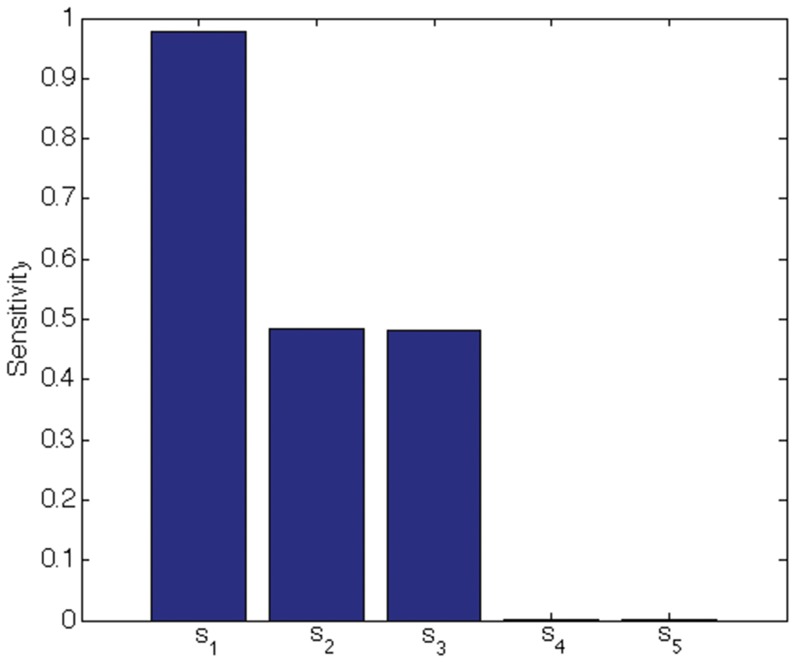
Class 1 sensitivity for toy data.

**Figure 19:**
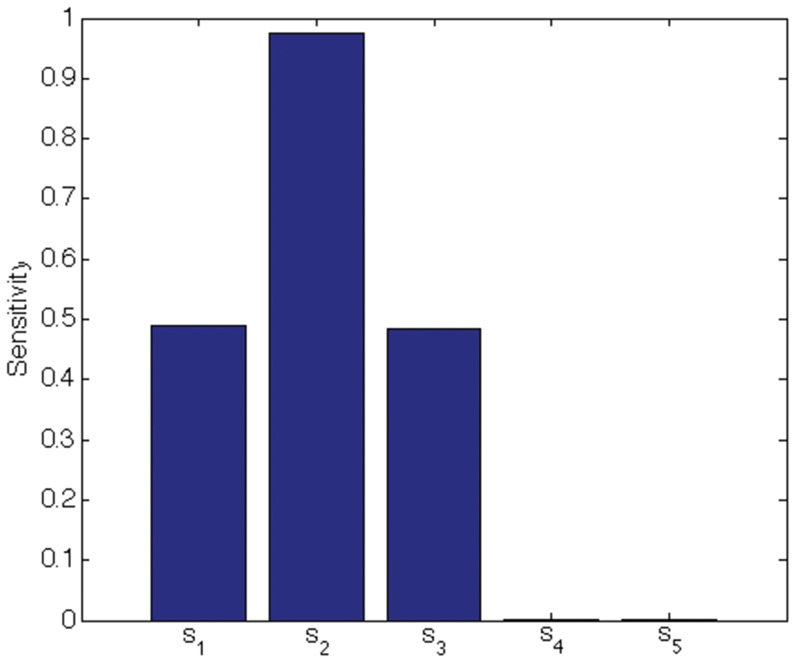
Class 2 sensitivity for toy data.

**Figure 20:**
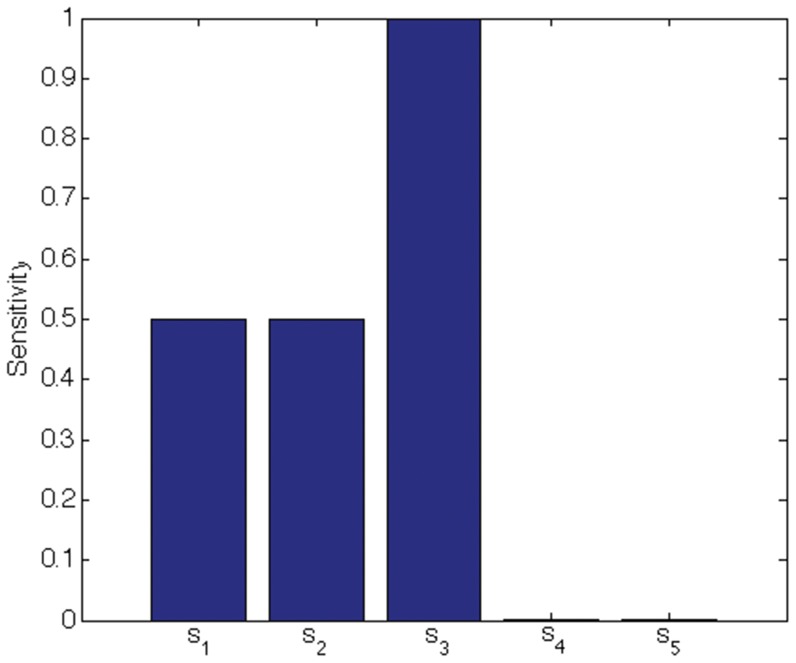
Class 3 sensitivity for toy data.

We indicate the usefulness of Scatter SVM sensitivity maps with two experiments. In the first experiment we create a five-dimensional training data set. Each dimension consists of 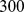 points, and is illustrated in Fig. 16. Only dimensions 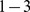 discriminate between three classes inherent in the data. We train the Scatter SVM such that it classifies a test set, generated in the same manner as the training set, perfectly. Fig. 17 shows the overall sensitivity coefficients 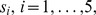 with respect to 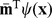. The result is in accordance with what to expect, namely that the Scatter SVM prediction function is sensitive to dimensions 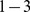. Sensitivity with respect to each class-wise prototype is shown in Figs. 18, 19 and 20.

**Figure 21:**
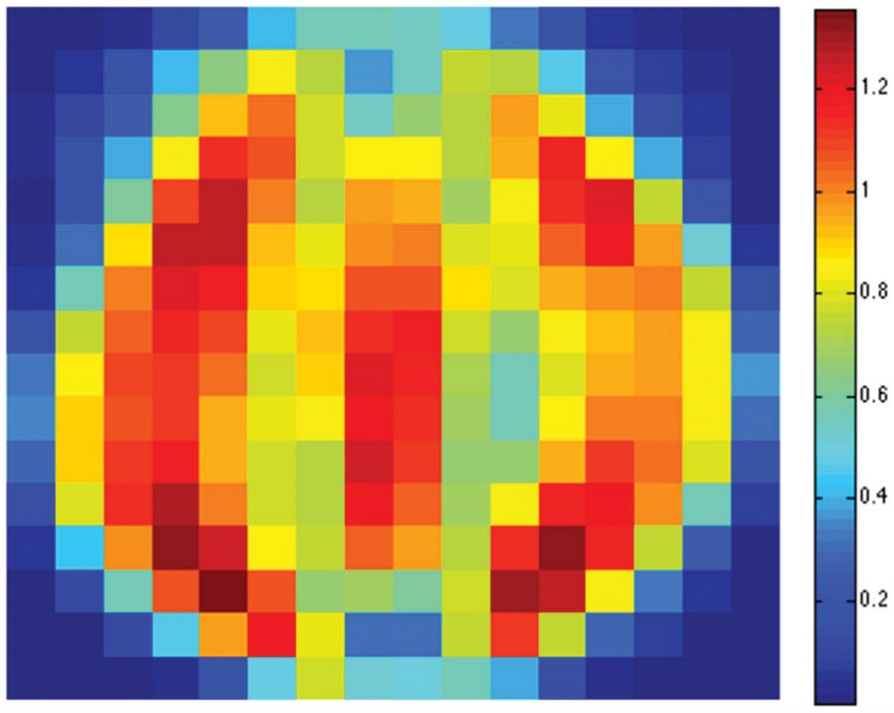
The overall sensitivity map for USPS digits “0”, “1” and “8”.

**Figure 22:**
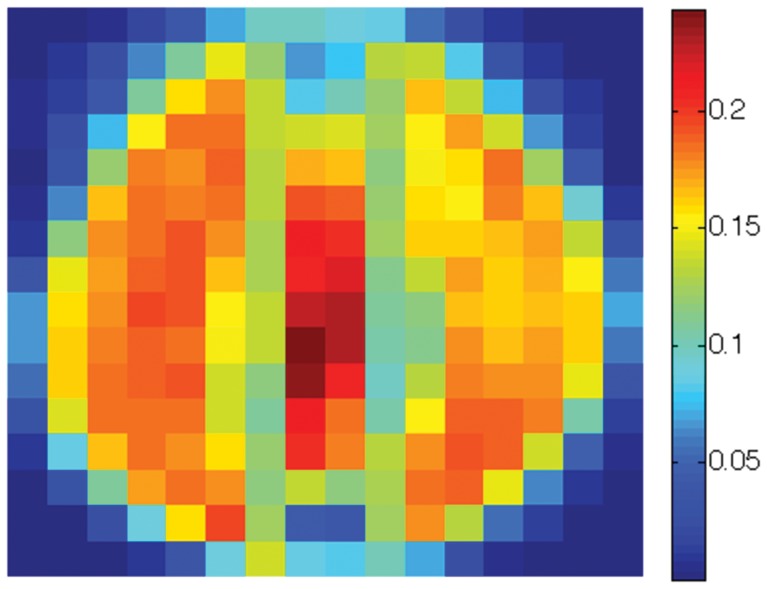
The sensitivity map corresponding to class “0” for USPS digits “0”, “1” and “8”.

**Figure 23:**
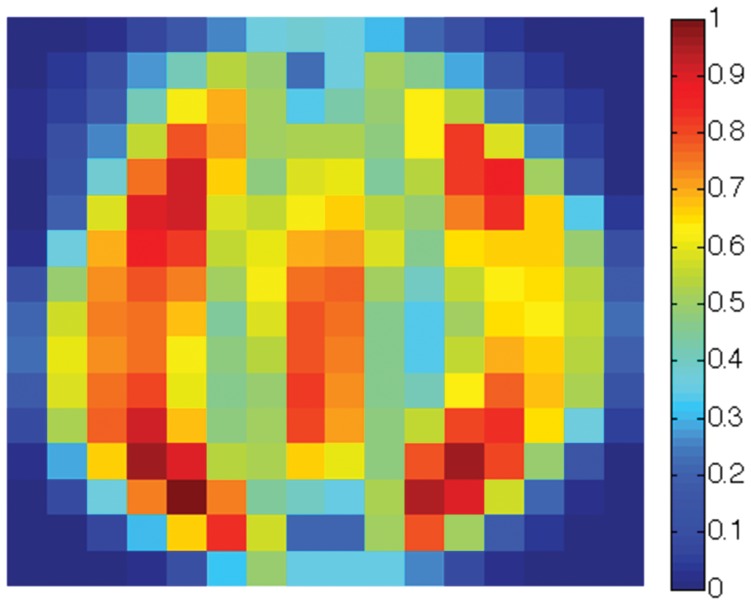
The sensitivity map corresponding to class “1” for USPS digits “0”, “1” and “8”.

**Figure 24:**
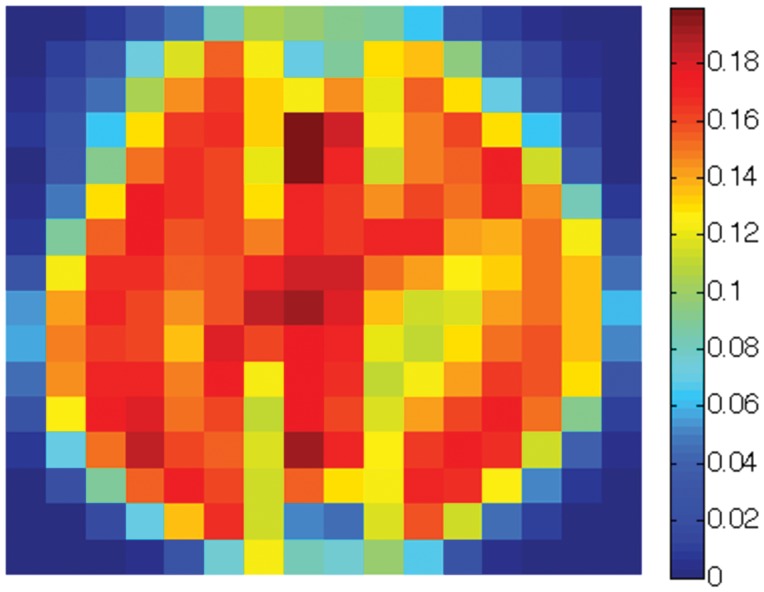
The sensitivity map corresponding to class “8” for USPS digits “0”, “1” and “8”.

Finally, we create a three-class data set consisting of the 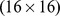 USPS digits “0”, “1” and “8” (a total of 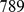 vectors of length 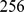), and train the Scatter SVM. When presenting the 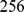 sensitivity weights, we rearrange them into a 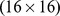 sensitivity map. The overall sensitivity map is shown in Fig. 21 (best viewed in colors). Note that the pixels near the boundaries show little sensitivity to the Scatter SVM classification. The most sensitive pixels are in regions which appear to be associated especially with the shapes of class “0” and class “1” digits. Figs. 22, 23 and 24 show the class-wise sensitivities for digits “0”, “1” and “8”, respectively, providing additional information.

We believe that Scatter SVM sensitivity maps may be especially useful for identifying active brain regions associated with some multi-category visual stimulus, or similar approaches, in neuroimaging. This we aim to study further in future work.

## Conclusions

We have in this exposition provided a novel prototype framework of the dual of 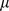-SVMs, involving the global mean of the prototypes as a key quantity in an interpretation based on the notion of scatter. This has enabled an extension to multiple classes, resulting in an optimization problem with a manageable number of variables and constraints. Furthermore, a full regularized risk minimization framework has been put forth for the primal problem, revealing the score function to be used in testing, and the role of the bias parameter. The Scatter SVM optimization problem has been implemented very efficiently in SHOGUN, thus offering a fast algorithm for multi-class classification. The results obtained are promising, also compared to the state of the art OVR, OVO and CS SVM implementations.

Intuitively, the Scatter SVM optimization problem enforces every sample to be *by a margin more similar to it's class mean than to the overall mean*. The assumptions under which our method will work well also becomes transparent, namely, there should be a certain *homogeneity* among the classes wrt. noise, outliers and regularization treatment. The reference to a global mean introduces a global regularization or stiffness of the model. There are, of course, learning problems that may require a fine grained class-wise regularization that is systematically only available by higher-dimensionally parameterized approaches like one vs. one. Note however that there is a sufficiently vast body of multi-class problems that match our assumptions above.

Furthermore, we have developed Scatter SVM multi-class sensitivity maps, and have indicated that useful visualization results are obtained.

Future work will exploit the Scatter SVM algorithm for image annotation, in subphoneme classification for speech recognition, computational biology and neuroinformatics. Visualization using sensitivity maps will in all these applications be useful for analysis and identification of discriminative features.

## Supporting Information

Appendix S1 **Primal Optimization Problem and Dualization.**
(PDF)

Appendix S2 **Shogun Implementation and Algorithm.**
(PDF)
