# Supplementary material for: A Scatter-Based Prototype Framework and Multi-Class Extension of Support Vector Machines
Source: PLoS One. 2012 Oct 30;7(10):e42947. doi: 10.1371/journal.pone.0042947 (PMC3484157; doi:10.1371/journal.pone.0042947)
Supplement: Appendix S1 — Primal Optimization Problem and Dualization. (PDF) [file pone.0042947.s001.pdf]

Appendix S1: Primal Optimization Problem and Dualization

The function $s(f, \mathbf{x}, y)$ provides a comparative measure between the scoring function $f_y(\mathbf{x})$ and $f_{c\neq y}(\mathbf{x})$ as input to the loss function, commonly defined in the literature as

$$s(f, \mathbf{x}, y) = f_y(\mathbf{x}) - \text{argmax}_{c\neq y}\ f_c(\mathbf{x}). \tag{1}$$

Because of the reference to the maximum of the scoring functions $f_{c\neq y}(\mathbf{x})$, a large number of constraints is introduced into the optimization problem. Much research has been aimed at solving Eq. (1) more efficiently, e.g. based on decomposition into smaller subproblems [1], interleaving column generation [2] or bundle methods [3], to name a few. But the limiting factor that the *max* represents, still remains.

As a remedy to this issue, we propose as a different and novel requirement that a hypothesis should score better than an *average* hypothesis, that is

$$s(f, \mathbf{x}, y) = f_y(\mathbf{x}) - \frac{1}{C}\sum_{c=1}^{C} f_c(\mathbf{x}).$$

For upcoming derivations, we focus on affine-linear models of the form

$$f_c(\mathbf{x}) = \mathbf{w}_c^\top \psi(\mathbf{x}) + b_c. \tag{2}$$

As discussed earlier, the bias parameter b_c may be removed in the derivations, which is a mild restriction for the high dimensional space $\mathcal{H}$ we consider. For the time being including the bias, the average hypothesis thus becomes $\bar{f}(\mathbf{x}) = \bar{\mathbf{w}}^\top \mathbf{x} + \bar{b}$ and

$$s(f, \mathbf{x}, y) = (\mathbf{w}_y - \bar{\mathbf{w}})^\top \psi(\mathbf{x}) + b_y - \bar{b}, \tag{3}$$

where $\bar{\mathbf{w}} = \frac{1}{C}\sum_{c=1}^{C} \mathbf{w}_c$ and $\bar{b} = \frac{1}{C}\sum_{c=1}^{C} b_c$. Each hyperplane $\mathbf{w}_c - \bar{\mathbf{w}}$, $c = 1, \ldots, C$, is associated with a margin ρ. The following quadratic regularizer aims to penalize the norms of these hyperplanes while at the same time maximizing the margins

$$\Omega(f) = \frac{1}{2}\sum_c \|\mathbf{w}_c - \bar{\mathbf{w}}\|^2 - C\rho. \tag{4}$$

The regularized risk thus becomes

$$\frac{1}{2}\sum_{c=1}^{C} ||\mathbf{w}_c - \bar{\mathbf{w}}||^2 - C\rho + \mu \sum_i l\left[(\mathbf{w}_{y_i} - \bar{\mathbf{w}})^\top \psi(\mathbf{x}_i) + b_{y_i} - \bar{b}\right].$$

Expanding the loss terms into slack variables leads to the *primal optimization problem*

$$\begin{array}{ll} \min\limits_{\mathbf{w}_c, \mathbf{w}, b, \rho, \mathbf{t}} & \frac{1}{2}\sum_c ||\mathbf{w}_c - \bar{\mathbf{w}}||^2 - C\rho + \mu \sum_i l(t_i) \\ s.t. & \langle \mathbf{w}_{y_i} - \bar{\mathbf{w}}, \psi(\mathbf{x}_i)\rangle + b_{y_i} \geq \rho - t_i, \quad \forall i \\ & \bar{\mathbf{w}} = \frac{1}{C}\sum_{c=1}^{C} \mathbf{w}_c \\ & \bar{b} = \frac{1}{C}\sum_{c=1}^{C} b_c = 0. \end{array} \tag{5}$$

The condition $\bar{b} = 0$ is necessary in order to obtain the primal of the binary μ-SVM as a special case of Eq. (5) and to avoid the trivial solution $\mathbf{w}_c = \bar{\mathbf{w}} = \mathbf{0}$ with $b_c = \rho \to \infty$.

The optimization problem Eq. (5) has an interesting interpretation. The constraint may be written as

$$\mathbf{w}_{y_i}^\top \psi(\mathbf{x}_i) + b_{y_i} \geq \bar{\mathbf{w}}^\top \psi(\mathbf{x}_i) + \rho - t_i.$$

Hence, we are learning for each class label a hyperplane function $f_{y_i}(\mathbf{x}) = \mathbf{w}_{y_i}^\top \psi(\mathbf{x}_i) + b_{y_i}$ that allows for scores better than the *average* by a margin. This is illustrated in Fig. 5 in the main manuscript.

Dualization

Optimization is often considerably easier in the dual space. As it will turn out, we can derive the dual problem of Eq. (5) without knowing the loss function l, instead it is sufficient to work with the Fenchel-Legendre dual $l^*(x) = \sup_t xt - l(t)$ (e.g. cf. [4,5]). Applying Lagrange's theorem incorporates the constraints into the objective by introducing non-negative Lagrangian multipliers $\boldsymbol{\alpha}, \boldsymbol{\beta}, \gamma$,

$$\begin{aligned}\mathcal{L} =& \frac{1}{2}\sum_c ||\mathbf{w}_c - \bar{\mathbf{w}}||^2 - C\rho + \mu \sum_i l(t_i) \\ &- \sum_i \alpha_i \left(\langle \mathbf{w}_{y_i} - \bar{\mathbf{w}}, \psi(\mathbf{x}_i) \rangle + b_{y_i} - \rho - t_i \right) \\ &+ \left\langle \boldsymbol{\beta}, \frac{1}{C}\sum_c \mathbf{w}_c - \bar{\mathbf{w}} \right\rangle + \gamma \sum_c b_c.\end{aligned}$$

Setting the partial derivatives of $\mathcal{L}$ wrt. the dual variables to zero gives the following optimality conditions,

$$\forall c: \ \mathbf{w}_c - \bar{\mathbf{w}} = \sum_{i:y_i=c} \alpha_i \psi(\mathbf{x}_i) - \frac{1}{C}\boldsymbol{\beta} \ ; \quad \mathbf{1}^\top \boldsymbol{\alpha}_c = 1 \ ,$$

where $\boldsymbol{\alpha} = \left(\boldsymbol{\alpha}_1^\top, ..., \boldsymbol{\alpha}_C^\top \right)^\top \in \mathbb{R}^n$ is equipped with a block structure and where $\boldsymbol{\alpha}_c^\top \mathbf{1} = \sum_{i:y_i=c} \alpha_i$. Note that the total number of variables in $\boldsymbol{\alpha}$ is thus n. Resubstituting into the Lagrangian and subsequently solving for $\frac{\partial \mathcal{L}}{\partial \boldsymbol{\beta}} = 0$ leads to

$$\begin{aligned}\forall c: \ \mathbf{w}_c &= \sum_{i:y_i=c} \alpha_i \psi(\mathbf{x}_i) \\ \bar{\mathbf{w}} &= \frac{1}{C}\sum_i \alpha_i \psi(\mathbf{x}_i); \quad \boldsymbol{\beta} = \sum_i \alpha_i \mathbf{x}_i.\end{aligned} \tag{6}$$

By the latter equations the Lagrangian saddle point problem can be expressed as

$$\sup_{\boldsymbol{\alpha}} \inf_t \quad -\frac{1}{2}\boldsymbol{\alpha}^\top \boldsymbol{\mathcal{K}} \boldsymbol{\alpha} + \mu \sum_i \left(l(t_i) + \alpha_i t_i \right), \tag{7}$$

$\boldsymbol{\alpha} : \boldsymbol{\alpha}^\top \mathbf{1} = C, \ \boldsymbol{\alpha}_c^\top \mathbf{1} = 1, \ c = 1, \ldots, C$ (if bias) where $\boldsymbol{\mathcal{K}}$ is given by Eq. (7) in the main manuscript. Inserting the notion of a Fenchel-Legendre dual function we can completely remove the dependency on the primal variables in Eq. (7), and obtain the generalized dual problem

$$\sup_{\boldsymbol{\alpha}} \quad -\frac{1}{2}\boldsymbol{\alpha}^\top \boldsymbol{\mathcal{K}} \boldsymbol{\alpha} - \mu \sum_i l^*(-\mu^{-1}\alpha_i), \tag{8}$$

$\boldsymbol{\alpha} : \boldsymbol{\alpha}^\top \mathbf{1} = C, \ \boldsymbol{\alpha}_c^\top \mathbf{1} = 1, c = 1, \ldots, C$ (if bias) where l^* is the Fenchel-Legendre conjugate function, which we subsequently denote as *dual loss* of l.

References

1. Crammer K, Singer Y (2001) On the Algorithmic Implementation of Multiclass Kernel-based Vector Machines. Journal of Machine Learning Research 2: 265-292.

2. Joachims T, Finley T, Yu CN (2009) Cutting-Plane Training of Structural SVMs. Machine Learning 77: 27-59.

3. Teo CH, Vishwanathan S, Smola A, Le Q (2010) Bundle Methods for Regularized Risk Minimization. Journal of Machine Learning Research 11: 311-365.

4. Boyd S, Vandenberghe L (2004) Convex Optimization. Cambridge, UK: Cambrigde University Press.

5. Smola AJ, Vishwanathan SVN, Le Q (2008) Bundle Methods for Machine Learning. In: Advances in Neural Information Processing Systems 20.
