# Supplementary material for: A Scatter-Based Prototype Framework and Multi-Class Extension of Support Vector Machines
Source: PLoS One. 2012 Oct 30;7(10):e42947. doi: 10.1371/journal.pone.0042947 (PMC3484157; doi:10.1371/journal.pone.0042947)
Supplement: Appendix S2 — Shogun Implementation and Algorithm. (PDF) [file pone.0042947.s002.pdf]

Appendix S2: Shogun Implementation and Algorithm

To describe the with-bias algorithm, we start from the 2-class ν-formulation as stated in Eq. (28) in [1] and repeated here

$$\inf_{\boldsymbol{\alpha'}} \quad \frac{1}{2}\boldsymbol{\alpha'}^\top \boldsymbol{\mathcal{K}}\boldsymbol{\alpha'}$$
$$\text{s.t} \quad \boldsymbol{y}^\top \boldsymbol{\alpha'} = 0, \quad \boldsymbol{\alpha'}^\top \mathbf{1} = \nu, \quad \mathbf{0} \leq \boldsymbol{\alpha'} \leq \frac{1}{N}\mathbf{1}.$$

We first notice that the two equality constraints can be expressed by class-wise total weight mass conditions: $\boldsymbol{\alpha'_1}^\top \mathbf{1} = \boldsymbol{\alpha'_{-1}}^\top \mathbf{1} = \nu/2$. Due to these equality constraints, reasonable subproblems require $y_i = y_j$; otherwise, neither α'_i nor α'_j could be changed. Consequently this constraint is implemented by the selection strategy and a proper choice of the initial solution candiate. Note that feasible initial points also require $\nu \leq C \cdot N_{min}/N$. To recover the problem in Eq. (15) in the main manuscript, we need to perform a variable transformation $\boldsymbol{\alpha'} \mapsto \frac{\boldsymbol{\alpha}}{\mu \cdot N}$ combined with the choice $\nu = C/(\mu \cdot N)$.

For 2-class problems, LIBSVM's working set selection strategy for ν-SVMs (cf. WSS 5 in [1]) traverses the active set twice and thus requires an effort of $\mathcal{O}(2N + 2T)$, where T is the time to compute a kernel row. A straightforward generalization traverses the active set for each of the C classes leading to an effort of $\mathcal{O}(CN + CT)$ which is what we used throughout experiments. However, when ordering examples, such that $y_i \leq y_j$ for $i < j$ and by creating C arrays to hold the maximum class-wise gradient etc. the computational complexity can be further reduced to $\mathcal{O}(C + N + CT)$.

We now describe our without-bias algorithm. We now face the problem that due to the lack of a bias there is no sum-to-one constraint on the α_i's anymore in the dual optimization problem, Eq. (15) in the main manuscript. Therefore the line search performed by SMO cannot be solved analytically anymore. As a remedy we implemented a without-bias solver based on SVMlight, which basically can deal with any quadratic programm. The algorithm is described in Algorithm 1. We thereby employ the notation $\boldsymbol{\mathcal{K}} = \boldsymbol{\kappa}(\mathbf{x}_i, \mathbf{x}_j)_{i,j=1}^n$ for the block kernel matrix as defined in Eq. (7) in the main manuscript.

The algorithm has as input an accuracy parameter ϵ (in our experiments $\epsilon = 0.001$ was chosen) and an active set size Q ($Q = 40$ was chosen). The main FOR loop (Lines 2-3) iterates until the stopping criterion (duality gap less than ϵ) is fulfilled. Line (a) computes the set of Q active variables based on minimal gradients. Line (b) performs the actual Scatter SVM computation w.r.t. the active variables, resulting in new values of the α_i. Line (c) updates the gradient w.r.t. the the new α_i. Line (d) computes the actual objective value of the optimization problem, Eq. (15) in the main manuscript.

Algorithm 1

1. $S^0 = -\infty$, $g_i = 0$, $\alpha_i = 0$, $\forall i = 1, ..., n$
2. **for** $t = 1, 2, \ldots$ and while optimality conditions are not satisfied, i.e. $|1 - \frac{S^t}{S^{t-1}}| \geq \epsilon$
   (a) Select Q variables $\alpha_{i_1}, \ldots, \alpha_{i_Q}$ based on the gradient $\mathbf{g}$ of Eq. (15) in the main manuscript, w.r.t. $\boldsymbol{\alpha}$
   (b) Store $\boldsymbol{\alpha}^{old} = \boldsymbol{\alpha}$ and then update $\boldsymbol{\alpha}$ according to Eq. (15) in the main manuscript, with respect to the selected variables
   (c) Update gradient $g_i \leftarrow g_i + \sum_{q=1}^{Q}(\alpha_{i_q} - \alpha_{i_q}^{old}) y_{i_q} \boldsymbol{\kappa}(\mathbf{x}_{i_q}, \mathbf{x}_i)$, $\forall\ i = 1, \ldots, n$
   (d) Compute the SVM objective $S^t = \sum_i y_i \alpha_i - \frac{1}{2}\sum_i y_i g_{m,i} \alpha_i$
3. **end for**

References

1. Fan RE, Chen PH, Lin CJ (2005) Working Set Selection Using the Second Order Information for Training SVM. Journal of Machine Learning Research 6: 1889–1918.
